# Orchidopexy for undescended testis—rate and predictors of re-ascent

**DOI:** 10.1007/s00383-024-05729-6

**Published:** 2024-05-28

**Authors:** Caroline Selin, Nilla Hallabro, Magnus Anderberg, Anna Börjesson, Martin Salö

**Affiliations:** 1 Department of Clinical Sciences, Pediatrics, Lund University, Lund, Sweden; 2 Department of Pediatric Surgery, Skåne University Hospital, 221 85 Lund, Sweden

**Keywords:** UDT, Orchidopexy, Re-ascent, Re-operation, Follow-up

## Abstract

**Purpose:**

This study aimed to investigate the rate of re-ascent requiring re-operation after primary orchidopexy and to investigate eventual differences between the inguinal and scrotal approach as well as other potential predictors for re-ascent.

**Methods:**

A retrospective cohort study of children treated for undescended testis (UDT) with orchidopexy between 2018 and 2022 was conducted. The primary outcome was re-ascent requiring re-operation, and the secondary outcome was atrophy rate. Independent variables were age, underlying conditions, side, surgical approach, operation time, bilaterality, congenital/ascended UDT, presence of scrotal hypoplasia, presence of a patent processus vaginalis, division of external oblique, and suture of the testis. Univariate and logistic regression were used to evaluate differences between groups and risk for re-ascent.

**Results:**

A total of 662 testes in 554 patients were included. Re-operation occurred in 6% (7% with inguinal approach, 3% with scrotal approach, *p* = 0.04). Re-operation was associated with younger age, congenital UDT, and inguinal approach, but neither of these variables remained significant in multivariate analyses. Atrophy occurred in one testis.

**Conclusion:**

The rate of re-ascent was 6% and the atrophy rate was 0.15%. A larger study may find predictors for re-ascent but with very low absolute risk. The lower rate of re-ascent with the scrotal approach is probably due to selection bias.

## Background

Undescended testis (UDT) is the most common urogenital malformation in newborns with a birth prevalence as high as 5% in some studies. After the spontaneous descent, a prevalence of 1.0–1.5% is often reported among 1-year-old boys [1]. During childhood, some boys present with ascending UDT resulting in a cumulative prevalence of UDT around 2% during childhood [2]. UDT is generally more common among boys born prematurely, small for gestational age, or with low birth weight [2]. Further, several genetic syndromes are associated with UDT, e.g., trisomy 21, Noonan, Prader–Willi, Klinefelter, etc. [3].

With the high prevalence of UDT and the possible late consequences in terms of decreased fertility and paternity rates, and increased risk of testicular cancer, correct and timely treatment with orchidopexy is crucial. Guidelines recommend orchidopexy between 6 and 12 months (Nordic) or < 18 months (European, American) [4–6]. Orchidopexy is performed through the traditional inguinal approach with or without division of the external aponeurosis, or through a scrotal approach. This latter technique was introduced as an alternative for the more distally placed UDTs but some use this for all palpable testes [7]. Whether or not to suture the tunica albuginea of the testis to the scrotal sac has been a subject of controversy. A meta-analysis with 1,736 patients included found higher rates of re-ascent in the group with patients subject to transscrotal suturing, probably reflecting that testes with tight funicles are more likely to be sutured [8].

In a 10-year-old review of outcomes following inguinal orchidopexy, re-ascent was seen in 4%, and atrophy of the testis was reported to occur in about 2% [9]. A recent prospective multicenter study has shown a re-ascent rate of 2.4% percent and an atrophy rate of 3% [10]. Similar results are reported in other articles as well [11–15] or with even lower re-ascent rates [12, 16]. The approach with a single scrotal incision has shown to have significantly shorter operation times but not lower re-operation or atrophy rates [17].

Thus, follow-up studies after orchidopexies have shown rather consistent numbers regarding atrophy rates but some variance regarding re-ascent that leads to re-operation. No recent study exists from the Nordic countries where a lower age for orchidopexy is recommended. Further, very few studies have reported possible predictors of re-ascent after orchidopexy. Hence, this study aimed to investigate the rate of re-ascent requiring re-operation after primary orchidopexy and to investigate eventual differences between inguinal and scrotal approaches as well as other potential predictors for re-ascent.

## Methods

Ethical approval (ref. no 2010/49) and approval from the hospital (ref. no 2019/11; regional permission to extract journal information) was obtained before the start of study.

### Study design

A retrospective cohort study of boys diagnosed and treated with (inguinal or scrotal) orchidopexy at a tertiary center of pediatric surgery and urology was performed. The center performs all operations for UDT in infants and all cases of non-palpable testis in a catchment area of around 2 million inhabitants. Orchidopexies for older boys with palpable testis are performed in a catchment area of around 1 million inhabitants. Generally, six surgeons operated on all cases during the study period.

### Exclusion and inclusion criteria

Patients were initially found using ICD-10 codes (Q53.1-2) and procedure code KFH00. Patients with palpable UDT who underwent orchidopexy between 2018 and 2022 were included. Also included were patients who at the initial out-patient examination were found to have a non-palpable UDT, but where the surgeon decided to advance with open orchidopexy, due to palpable UDT under general anesthesia, diagnostic laparoscopy findings, imaging, or other reasons (*n* = 86 testes). However, if exploration revealed an atrophied testis or if an orchiectomy was performed for any other reasons, the patient was excluded.

Re-operations referred from other centers were excluded, as were patients previously operated for ipsilateral inguinal hernia. Patients lacking follow-up were also excluded. Ambulatory patients with a suspected strangulated inguinal hernia were excluded, as well as patients with a suspected undescended testicular torsion that were acutely explorated, even if orchidopexy was performed in the same procedure.

### Outcomes

The primary outcome was re-ascent of the testis which led to a re-operation. The secondary outcome was the rate of atrophy.

### Independent variables

The medical and surgical records were used to extract all variables; age at surgery, prematurity, genetic syndrome, laterality, bilaterality, congenital or ascended UDT, scrotal hypoplasia, inguinal or scrotal approach, whether the external oblique was opened, whether the testis was sutured to the scrotal sac, presence of patent processus vaginalis, and operation time.

### Definitions

A genetic syndrome was defined as a syndrome caused by a chromosomal abnormality (e.g., trisomi 21, karyotype XXY etc.) or a mutation/genetic polymorphism in a specific gene locus with previously known connected comorbidities or malformations. Prematurity was defined as delivery before week 37 + 0. Whether the UDT was acquired or congenital was concluded from information in medical records (including information from the parents as well as information in the referral letter regarding UDT). If still not concludable, children older than 2 years old at referral, and born in Sweden, were assumed to have been to well-child check-ups (five scheduled routine exams between 6 and 18 months old, where the testes should be routinely examined ) and the UDT was considered acquired. In cases where the child was not born in Sweden or medical record data were conflicting, information on congenital or acquired UDT was left blank. Cases where a scrotal approach first was taken, but surgery revealed a need for an inguinal approach, were classified as inguinal. In cases where both testes had been operated in the same procedure, the time was divided by two to get the mean operation time for each testis. In cases where other surgical procedures had been performed during the same operation, the time was left blank. Unilateral undescended testis was defined as a contralateral testis in a scrotal position or considered retractile at the time of surgery. Bilateral undescended was defined as a contralateral testis previously operated on or not scrotally placed at the time of surgery. Scrotal hypoplasia and a patent processus vaginalis was considered to be present when this clearly was described in the medical journal or operative notes. The testis was considered atrophied when medical records from the check-ups stated that it had undergone atrophy.

### Statistical analysis

All statistical analyses were performed with SPSS (IBM SPSS Statistics for Windows, Version 27.0. Armonk, NY). The significance level was set at 0.05. Since data were normally distributed, the student’s *t* test was used when comparing groups with a continuous variable. When comparing categorized data with binary outcomes, the Chi2 test was used. Logistic regression was performed to evaluate risk and possible predictors for re-ascent and presented with odds ratio (OR) and adjusted OR (aOR) with 95% confidence intervals (95%CI). Since re-ascent was a rather uncommon outcome, only significant variables from the univariate analysis were further explored in a multivariate analysis.

## Results

**Fig. 1:**
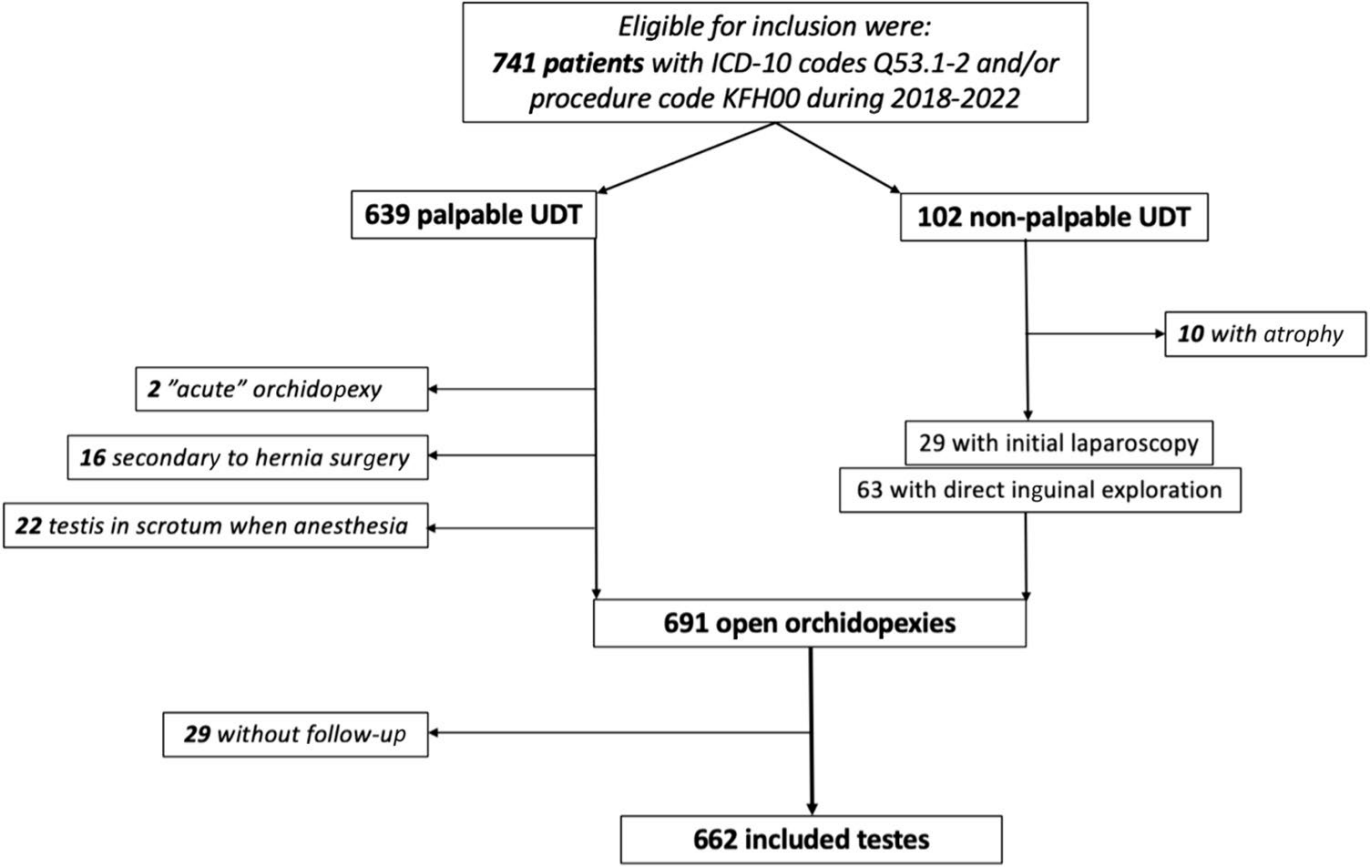
Flowchart visualizing the process of inclusion and exclusion of testes with a total of 662 testes undergoing orchidopexy included in total. *UDT* undescended testis

Seven hundred forty-one cases of UDT were found to match the inclusion criteria, with either palpable UDT (*n* = 639) or non-palpable but underwent open orchidopexy, either due to diagnostic laparoscopy, palpable UDT under general anesthesia or other reasons (*n* = 102). After the exclusion of testes with atrophy, “acute” orchidopexy, UDT secondary to previous inguinal hernia surgery, testis in scrotal position under general anesthesia or patients lost to follow-up, there were finally 662 testes in 554 boys included. A total of 109 patients had bilateral surgery, and of those, 92 had bilateral surgery during the same procedure (Fig. 1).

**Fig. 2:**
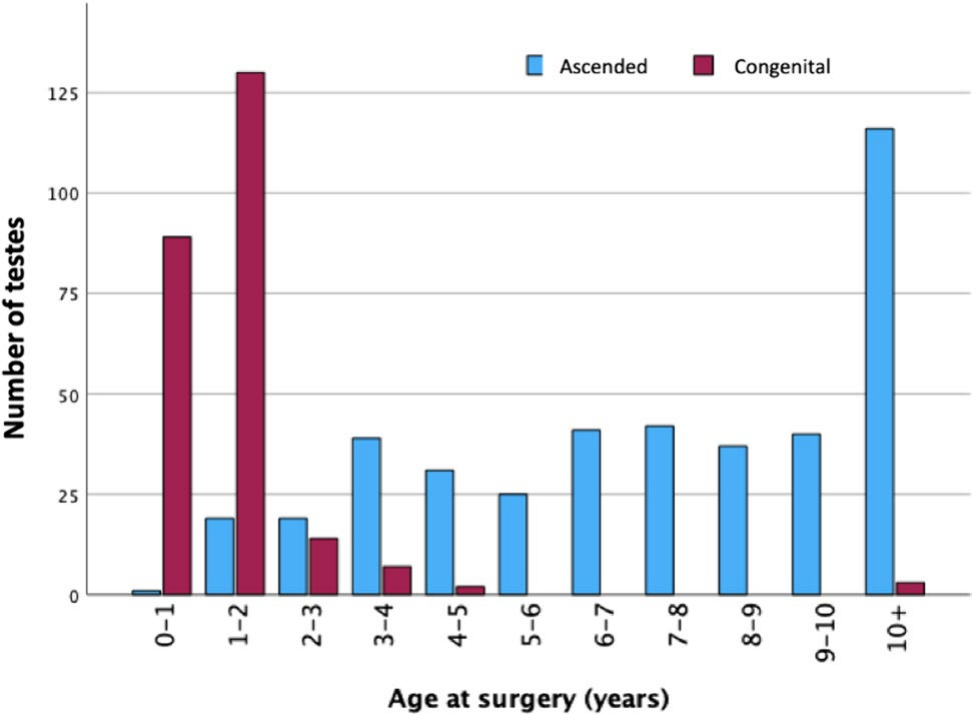
Age in years at the surgery of each testis in 662 boys operated on with orchidopexy. Red bar: congenital undescended testes; blue bar: ascended testes. (Missing data: *n* = 7)

In total, 37% (*n* = 245) of all operated testes were congenital UDTs, 63.0% were ascended, and 36.3% had bilateral disease. A total of 70.2% of all testes were operated on by an inguinal approach (Table 1). Three scrotal operations were converted to an inguinal approach, and these testes did not re-ascend or atrophy. Two main different sorts of scrotal incision were used; most often a transverse scrotal stria incison, but in around 10%, a lateral scrotal incision was used depending on patient anatomy and preference of the surgeon. The majority of all congenital UDTs were operated on when the patients were between 1 and 2 years, and the largest number of ascended UDTs were found in the group of 10 years and older (age at surgery) (Fig. 2).

**Table 1:** Comparison between testes operated on with inguinal or scrotal approach regarding demographics, pathogenesis, and surgical factors at primary surgery

	Total (*n* = 662)	Inguinal approach (*n* = 469)	Scrotal approach (*n* = 193)	*p* value
Age at surgery (months)	63.7 (± 48.2)	61.3 (± 49.9)	69.6 (± 43.4)	0.033*
Bilaterality	240 (36.3%)	137 (29.2%)	103 (53.4%)	< 0.001**
Genetic syndrome	30 (4.5%)	29 (6.2%)	1 (0.52%)	0.001**
Premature	36 (5.4%)	29 (6.2%)	7 (3.6%)	0.187*
Congenital	245 (37.0%)	207 (44.1%)	38 (19.7%)	< 0.001**
Right side	375 (56.6%)	259 (55.2%)	116 (60.1%)	0.250*
Sutured to scrotal sac	316 (47.8%)	290 (61.8%)	26 (13.5%)	< 0.001**
External oblique divided	369 (55.7%)	369 (78.7%)	0	N/A
Operation time (min)^a^	41.4 (± 19.4)	48.3 (± 18.1)	25.5 (± 11.4)	< 0.001**
Patent processus vaginalis	368 (55.6%)	284 (60.1%)	84 (43.5%)	< 0.001**
Scrotal hypoplasia	189 (28.5%)	136 (29.0%)	53 (27.5%)	0.691**

Values presented as mean (± SD) and the absolute number and percentage of patients

^a^38 patients with missing data

*Student’s *t* test, **Chi-square

Patients operated on with an inguinal approach were significantly younger, and more often had genetic syndromes or congenital UDT as compared to the patients operated on with the scrotal approach. They were also more likely to have a patent processus vaginalis. Those operated with a scrotal approach more often had bilateral disease, but were less likely to have the testis sutured in the scrotum. There were no significant differences in the rate of prematurity, side or scrotal hypoplasia between the different approaches (Table 1).

In total, 40 out of 662 (6%) of all orchidopexies required re-operation due to re-ascent. Re-ascent occurred in 34 testes (7.2%) that had undergone surgery by inguinal approach, and 6 (3.1%) of testes scrotally approached (*p* = 0.042). Only one (0.15%) testis had atrophied after orchidopexy (inguinal operation). The mean time of surgery was 48.3 (SD = 18.1) min for the inguinal orchidopexies and 25.5 (SD = 11.4) min for the scrotal orchidopexies (*p* < 0.001) (Table 2).

**Table 2:** Outcomes in terms of atrophy, re-ascent, and mean operation time after surgery in undescended testes operated on with an inguinal or scrotal approach

	Total (*n* = 662)	Inguinal approach *n* = 469 (70.8%)	Scrotal approach *n* = 193 (29.2%)	*p* value
Atrophy	1 (0.15%)	1 (0.2%)	0 (0.0%)	0.119*
Re-ascent	40 (6.0%)	34 (7.2%)	6 (3.1%)	0.042*
Operation time (min)	41.4 (± 19.4)	48.3 (± 18.1)	25.5 (± 11.4)	< 0.001**

Values presented as the absolute number and percentage of patients, *n* (%), and as mean (± standard deviation)

*Chi-square, **Student’s *t* test

When comparing the testes with and without re-ascent at follow-up requiring re-operation, there were no significant differences regarding the presence of genetic syndromes, prematurity, laterality, bilaterality, patent processus vaginalis, scrotal hypoplasia or mean operation time. An inguinal approach was significantly more common in the group requiring re-operation (*p* = 0.042), but whether the external oblique aponeurosis had been divided or not at the primary operation did not differ between the groups. The group requiring re-operations had a significantly lower mean age, a higher rate of congenital UDT, and were more often operated on by an inguinal approach (Table 3). Neither of these three variables remained significant in a multivariate logistic regression; age at surgery aOR = 0.989 (95% CI = 0.976–1.002), congenital UDT aOR = 1.093 (95% CI = 0.374–3.197), and inguinal approach aOR = 1.997 (95% CI = 0.791–5.043) (Table 4).

**Table 3:** Differences between undescended testes requiring re-operation and not regarding demographics, pathogenesis, and surgical factors at primary surgery

	No re-operation (*n* = 622)	Re-operation (*n* = 40)	*p* value
Age at surgery (months)	65.1 (± 48.4)	42.3 (± 40.2)	0.001*
Bilaterality	226 (36.3%)	14 (35%)	0.865**
Genetic syndrome	29 (4.7%)	1 (2.5%)	0.524**
Premature	34 (5.5%)	2 (5%)	0.900**
Congenital	222 (35.7%)	23 (57.5%)	0.002**
Right side	358 (57.6%)	17 (42.5%)	0.063**
Inguinal approach	435 (69.9%)	34 (85%)	0.042*
Divided external oblique	346 (55.6%)	23 (57.5%)	0.817**
Sutured to scrotal sac	299 (48.1%)	17 (42.5%)	0.494**
Operation time (min)	41.2 (± 19.3)	41.5 (± 20.4)	0.187*
Patent processus vaginalis	341 (54.8%)	27 (67.5%)	0.118
Scrotal hypoplasia	177 (28.5%)	12 (30.0%)	0.834

Values presented as mean (± SD) and the absolute number and percentage of patients

*Student’s *t* test, **Chi-square

**Table 4:** Univariate and multivariate analysis of potential risk factors for re-ascent after orchidopexy of 662 palpable undescended testes

	OR [95% CI]	*p* value	aOR [95% CI]	*p* value
Age at surgery (months)	0.989 [0.981–0.997]	0.005	0.989 [0.976–1.002]	0.085
Bilaterality	1.060 [0.542–2.071]	0.865		
Genetic syndrome	0.525 [0.070–3.951]	0.531		
Prematurity	0.910 [0.211–3.932]	0.900		
Congenital	2.728 [1.395–5.337]	0.003	1.093 [0.374–3.197]	0.871
Right side	1.835 [0.961–3.503]	0.066		
Inguinal approach	2.436 [1.006–5.900]	0.049	1.997 [0.791–5.043]	0.143
Divided external oblique	1.079 [0.565–2.060]	0.817		
Sutured to scrotal sac	0.798 [0.418–1.524]	0.495		
Operation time (min)	1.011 [0.995–1.027]	0.188		
Scrotal hypoplasia	1.077 [0.536–2.166]	0.834		
Patent processus vaginalis	1.711 [0.867–3.379]	0.122		

*OR* odds ratio, *CI* confidence interval, *aOR* adjusted OR

*Significant *p* values < 0.05

## Discussion

This retrospective cohort study of nearly 700 testes which underwent orchidopexy found a total rate of re-ascent of 6%. Patients who were re-operated were significantly younger, more often had congenital UDT, and were more often operated on with an inguinal approach. However, neither of these variables remained significant in adjusted analyses. Atrophy occurred only in one testis. As expected, operation times were significantly longer and re-ascent more common in boys operated with an inguinal compared to a scrotal approach.

Previous studies have reported very different rates of re-ascent after surgery for UDT, from 1% up to as much as 10% [9–16, 18]. Most studies report a rate of around 2–3% and the rate of 6% concluded from this study is, thus, higher than most, but not all, studies. Reasons behind this can be differences in surgical technique and/or expertise, different definitions of UDT and perhaps especially the difference between ascending and retractile testicles, different types and time to follow-up, and different definitions of a correct position of the testis post orchidopexy. Since this study showed a much lower atrophy rate compared to previous studies (discussed below), one speculation is that a more aggressive dissection of the cord structures may lead to a lower rate of re-ascent but a higher rate of atrophy.

The re-ascent rates differed significantly between the different approaches (inguinal 7.2%; scrotal 3.1%), but the approach was not found to be a significant predictor of re-ascent. Re-ascent was also significantly more common among congenital UDTs than ascended UDTs, and the mean age was significantly lower in the re-ascent group, but neither of those factors was found to predict for re-ascent in the multivariate logistic regression analysis. Not finding any statistically significant predictors for the need for re-operation in a multivariate regression might be due to the lack of statistical power rather than actual reality. Since only 40 testes re-ascended (6%), the outcome is quite uncommon, and may therefore require a larger cohort population. However, even if there would be a statistically significant difference, the effect and clinical relevance would be small.

In this study, the rate of re-ascent after surgery for UDT was twice as high after an inguinal approach as compared to a scrotal approach, even if the surgical approach did not fall out as a significant predictor in the multivariate analysis.

This probably reflects the choice of an inguinal approach in tight and short structures (high inguinal UDT), since it is only possible to do further mobilization retroperitoneal and divide the external oblique through an inguinal approach [19]. Some surgeons exclusively choose a scrotal approach in UDTs close to the scrotum, and one may suspect that those testes are not the most prone to re-ascent [19]. Hence, the exact location of the testis (centimeters away from the scrotum) could probably be an important factor in calculating the risk of re-ascent. Another factor not included in this study could be surgical experience.

The rate of atrophy was much lower in this study (only one of 662 testes; 0.15%) than previously reported rates of approximately 2% [9, 10, 12]. This may reflect the skill of the surgeons, or at least variation in how aggressively the chord structures are dissected, as discussed above. In this study, mainly 6 surgeons performed nearly all 662 orchidopexies during the 5 years reviewed, which reflects the general trend toward sub-specialization in general [20]. Another possible explanation for different atrophy rates is variations in the management of atrophied or dysplastic testes discovered during the planned orchidopexy. In this study, ten testes were directly extirpated, due to atrophy or what was thought to be severe dysplasia. Assuming all these were to be operated on and later removed, a rate of nearly 2% would be acquired. A methodological weakness in this study is the definition of atrophy, where a testis only was considered atrophied when stated so by medical records. A more objective measurement of atrophy to use in future studies may be shrinkage from its pre-operative size, but due to technical limitations, that definition was not eligible in this study.

Regarding operation times, there were statistically significant differences between inguinal and scrotal approaches, with a mean of 48 and 26 min, respectively. This is expected and in line with previous reports [14] of shorter operation times for the scrotal approach. The inguinal approach was more often chosen in patients with genetic syndromes, congenital UDTs, and unilateral UDT, and had a lower mean age at surgery.

This study will add important data to the outcome after orchidopexy since the study is rather large, and has a somewhat higher re-ascent rate but a much lower atrophy rate compared to previous studies. It is also one of very few studies evaluating predictors for re-ascent. Another strength is the low number lost to follow-up (*n* = 29) and that one person collected all data, reducing systematic errors of different interpretations. The study, of course, also has limitations that need to be regarded, all mainly reflecting the retrospective design. First, there is an inherent bias in the selection of an inguinal or scrotal approach, since surgeons probably use the scrotal approach for more distal UDT. The only way to avoid this bias is through a randomized controlled trial. We are, however, of the opinion that “high” UDT should be operated through an inguinal incision with a division of the external oblique. Another limitation was the assumptions made in unclear cases whether the UDT was congenital or ascended since this assumes a sufficient competence of the doctors in well-child check-ups. However, there were cases found when reading journal notes for this study where the physician did not examine the testes properly or did not refer the child at the recommended time. Thus, more UDTs than assumed in the paper may be congenital. In this study, 63% of all orchidopexied testes were found to be ascended, which is somewhere in between previous studies, reporting rates from 17.9 to 74% of all UDTs [21, 22].

In future research, potentially interesting variables to further evaluate as risk factors for re-ascent would be surgical experience [10], BMI of the child, a more exact description of the location of the UDT, and some kind of objective measurement of how tense cord structures are. Since orchidopexy is one of the most common surgeries performed on children, even small improvements in surgical techniques can benefit many boys and their parents as well as reduce costs.

## Conclusion

Six percent of all orchidopexied testes needed a re-operation due to re-ascent after primary orchidopexy. The inguinal approach had double the rate of re-ascent compared to the scrotal approach but neither this nor other predictors of re-ascent remained significant in multivariate analyses. Atrophy was very rare. The scrotal approach had as expected significantly shorter operation times.

## Data Availability

No datasets were generated or analyzed during the current study.

## References

[1] Sijstermans K, Hack WWM, Meijer RW, Voort-Doedens LVD (2008) The frequency of undescended testis from birth to adulthood: a review. Int J Androl 31(1):1–11 17488243 10.1111/j.1365-2605.2007.00770.x

[2] Bergbrant S, Omling E, Björk J, Hagander L (2018) Cryptorchidism in sweden: a nationwide study of prevalence, operative management, and complications. J Pediatr 194:197-203.e6 29331326 10.1016/j.jpeds.2017.09.062

[3] Foresta C, Zuccarello D, Garolla A, Ferlin A (2008) Role of hormones, genes, and environment in human cryptorchidism. Endocr Rev 29(5):560–580 18436703 10.1210/er.2007-0042

[4] Kolon TF, Herndon CD, Baker LA, Baskin LS, Baxter CG, Cheng EY et al (2014) Evaluation and treatment of cryptorchidism: AUA guideline. J Urol 192(2):337–345 24857650 10.1016/j.juro.2014.05.005

[5] Martin Ritzén E, Bergh A, Bjerknes R, Christiansen P, Cortes D, Haugen S et al (2007) Nordic consensus on treatment of undescended testes. Acta Paediatr 96(5):638–643 17326760 10.1111/j.1651-2227.2006.00159.x

[6] Radmayr C, Bogaert G, Burgu B, Castagnetti MS, Dogan HS, O’Kelly F et al (2023) EAU guidelines on paediatric urology 2023. EAU Guidel Paediatr Urol 2023:14–19

[7] Bassel YS, Scherz HC, Kirsch AJ. Scrotal incision orchiopexy for undescended testes with or without a patent processus vaginalis. J Urol. 2007;177(4):1516-8. 10.1016/j.juro.2006.11.075 17382769

[8] Anand S, Singh A, Bajpai M (2021) Transparenchymal testicular suture: a systematic review and meta-analysis highlighting the impact of additional fixation suture during routine orchiopexy. J Pediatr Urol 17(2):183–189 33478901 10.1016/j.jpurol.2020.12.012

[9] Penson D, Krishnaswami S, Jules A, McPheeters ML (2013) Effectiveness of hormonal and surgical therapies for cryptorchidism: a systematic review. Pediatrics 131(6):e1897–e1907 23690511 10.1542/peds.2013-0072 PMC4074661

[10] Timing of orchidopexy and its relationship to postoperative testicular atrophy: results from the ORCHESTRA study. BJS Open. 2021;5(1). 10.1093/bjsopen/zraa052 PMC7893476 33609392

[11] Docimo SG (1995) The results of surgical therapy for cryptorchidism: a literature review and analysis. J Urol 154(3):1148–1152 7637073

[12] Durell J, Johal N, Burge D, Wheeler R, Griffiths M, Kitteringham L et al (2016) Testicular atrophy following paediatric primary orchidopexy: a prospective study. J Pediatr Urol 12(4):243.e1–4 27422375 10.1016/j.jpurol.2016.05.023

[13] McIntosh LA, Scrimgeour D, Youngson GG, Driver CP (2013) The risk of failure after primary orchidopexy: an 18 year review. J Pediatr Urol 9(6 Pt A):759–762 23032098 10.1016/j.jpurol.2012.09.002

[14] Neheman A, Levitt M, Steiner Z (2019) A tailored surgical approach to the palpable undescended testis. J Pediatr Urol 15(1):59.e1-e5 30563750 10.1016/j.jpurol.2018.08.022

[15] Noseworthy J (2003) Recurrent undescended testes. Semin Pediatr Surg 12(2):90–93 12728393 10.1016/s1055-8586(02)00017-3

[16] Meij-de Vries A, Goede J, van der Voort L, Heij HA, Meijer RW, Hack WW (2012) Long-term testicularposition and growth of acquired undescended testis after prepubertal orchidopexy. J Pediatr Surg. 47(4):727–35 22498388 10.1016/j.jpedsurg.2011.10.073

[17] Feng S, Yang H, Li X, Yang J, Zhang J, Wang A et al (2016) Single scrotal incision orchiopexy versus the inguinal approach in children with palpable undescended testis: a systematic review and meta-analysis. Pediatr Surg Int 32(10):989–995 27510940 10.1007/s00383-016-3956-4

[18] Maizels M, Gomez F, Firlit CF (1983) Surgical correction of the failed orchiopexy. J Urol 130(5):955–957 6138441 10.1016/s0022-5347(17)51594-3

[19] Barthold J, Hagerty J (2021) Etiology, diagnosis and management if the undescended testis. In: Partin A, Dmochowski R, Kavoussi L, Peters C (eds) Campbell–Walsh–Wein urology, 12th edn. Elsevier, Philadelphia, pp 949–972

[20] Longo WE, Sumpio B, Duffy A, Seashore J, Udelsman R (2008) Early specialization in surgery: the new frontier. Yale J Biol Med 81(4):187–191 19099049 PMC2605312

[21] Hack WWM, Meijer RW, Van Der Voort-Doedens LM, Bos SD, De Kok ME (2003) Previous testicular position in boys referred for an undescended testis: further explanation of the late orchidopexy enigma? BJU Int 92(3):293–296 12887487 10.1046/j.1464-410x.2003.04317.x

[22] Wohlfahrt-Veje C, Boisen KA, Boas M, Damgaard IN, Kai CM, Schmidt IM et al (2009) Acquired cryptorchidism is frequent in infancy and childhood. Int J Androl 32(4):423–428 19515170 10.1111/j.1365-2605.2008.00946.x

